# Supplementary material for: Causal Relationship between Aging and Anorexia Nervosa: A White-Matter-Microstructure-Mediated Mendelian Randomization Analysis
Source: Biomedicines. 2024 Aug 16;12(8):1874. doi: 10.3390/biomedicines12081874 (PMC11351342; doi:10.3390/biomedicines12081874)
Supplement: Supplementary file 1 [file biomedicines-12-01874-s001.zip › Supplementary Figure/Figure S4/Singe SNP plot of MR analysis for TL to ubm-b-1766.pdf]

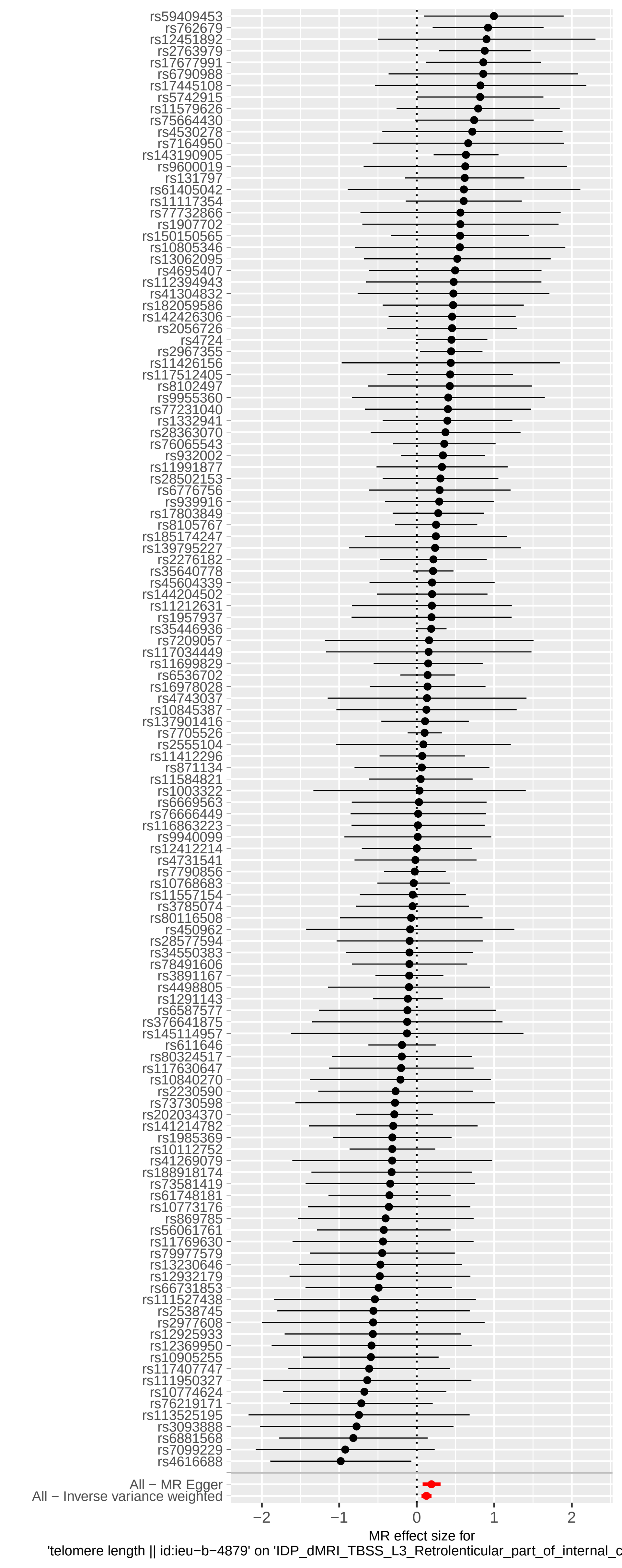

rs59409453
rs762679
rs12451892
rs2763979
rs17677991
rs6790988
rs17445108
rs5742915
rs11579626
rs75664430
rs4530278
rs7164950
rs143190905
rs9600019
rs131797
rs61405042
rs11117354
rs77732866
rs1907702
rs150150565
rs10805346
rs13062095
rs4695407
rs112394943
rs41304832
rs182059586
rs142426306
rs2056726
rs4724
rs2967355
rs11426156
rs117512405
rs8102497
rs9955360
rs77231040
rs1332941
rs28363070
rs76065543
rs932002
rs11991877
rs28502153
rs6776756
rs939916
rs17803849
rs8105767
rs185174247
rs139795227
rs2276182
rs35640778
rs45604339
rs144204502
rs11212631
rs1957937
rs35446936
rs7209057
rs117034449
rs11699829
rs6536702
rs16978028
rs4743037
rs10845387
rs137901416
rs7705526
rs2555104
rs11412296
rs871134
rs11584821
rs1003322
rs6669563
rs76666449
rs116863223
rs9940099
rs12412214
rs4731541
rs7790856
rs10768683
rs11557154
rs3785074
rs80116508
rs450962
rs28577594
rs34550383
rs78491606
rs3891167
rs4498805
rs1291143
rs6587577
rs376641875
rs145114957
rs611646
rs80324517
rs117630647
rs10840270
rs2230590
rs73730598
rs202034370
rs141214782
rs1985369
rs10112752
rs41269079
rs188918174
rs73581419
rs61748181
rs10773176
rs869785
rs56061761
rs11769630
rs79977579
rs13230646
rs12932179
rs66731853
rs111527438
rs2538745
rs2977608
rs12925933
rs12369950
rs10905255
rs117407747
rs111950327
rs10774624
rs76219171
rs113525195
rs3093888
rs6881568
rs7099229
rs4616688
All − MR Egger
All − Inverse variance weighted
−2
−1
0
1
2
MR effect size for
'telomere length || id:ieu−b−4879' on 'IDP_dMRI_TBSS_L3_Retrolenticular_part_of_internal_c
